# When should a premature neonate (24–35 weeks) be discharged? five-year experience from a single-center neonatal intensive care unit in Türkiye

**DOI:** 10.55730/1300-0144.5690

**Published:** 2023-05-03

**Authors:** Ayşen ORMAN, Yalçın ÇELİK, Semra ERDOĞAN

**Affiliations:** 1 Division of Neonatology, Department of Pediatrics, Faculty of Medicine, Mersin University, Mersin, Turkiye; 2 Department of Biostatistics and Medical Informatics, Faculty of Medicine, Mersin University, Mersin, Turkiye

**Keywords:** Preterm neonate, hospital length of stay, discharge

## Abstract

**Background/aim:**

The survival rate among preterm infants has improved, and hospital stays have been prolonged, consistent with positive developments in perinatal and neonatal care. The aim of this study was to provide evidence-based information for healthcare professionals concerning the ideal time for discharge by evaluating the reasons for prolonged hospital stays.

**Materials and methods:**

Six hundred eighty-one premature babies born at 24–35 weeks at the Mersin University Medical Faculty Hospital between January 2016 and May 2020 and admitted to the neonatal intensive care unit were included in the study following a retrospective file examination. Date of birth (gestational age) and discharge week (duration of hospital stay) calculated from the date of final discharge were recorded. Based on the literature, the ideal discharge time was determined to be 40 weeks according to postmenstrual age (week of birth + length of hospital stay). The primary variable was whether the infants were discharged before the ideal discharge week. The secondary variable was the effect of the presence of comorbidity on the length of hospital stay and ideal discharge time.

**Results:**

The mean hospital stay of preterm neonate born at 25^0–7^–26^0–7^, 27^0–7^–28^0–7^ and 29^0–7^–30^0–7^ weeks was significantly shorter in the absence of comorbidity than in the presence of comorbidity (p = 0.001, 0.004, and 0.008, respectively). More than half (53.5%) were discharged before the expected date of birth as gestational weeks increased, despite the prolonged length of stay in the presence of comorbidity.

**Conclusion:**

Health professionals can inform families that, in the absence of comorbidity, discharge is possible at an average of 36 weeks for 25^0–7^–28^0–7^-week gestational ages, and at an average of 34 weeks for 29^0–7^–32^0–7^-week gestational ages.

## Introduction

Prematurity and related comorbidities are important reasons for admission to neonatal intensive care. Following advances in science and technology, the survival rate of preterm infants has increased, and the length of stay (LOS) in the neonatal intensive care unit (NICU) has become particularly important. Although the ideal discharge times for premature infants are described by the expected time of birth or when they can be fed and keep themselves warm, these criteria cannot be applied to every case. However extremely preterm (<28 weeks) and late preterm (34^0–7^–36^0–7^) infants may not have the same severe medical problems. This causes differences in discharge times due to LOS. Discharging preterm infants as soon as possible has long been known to strengthen and support the bond between mother and infant [1]. Other benefits of early discharge involve hospital costs, lower health care-associated infection risks, less exposure to adverse effects of light and noise, and more beds and care time being available for acute patients [2]. Data concerning the length of hospital stay of these infants are important in terms of informing parents about prognosis and time of discharge. In this study, each gestational week among infants born at weeks 24–35 was evaluated separately in terms of discharge risk factors.

Few studies in the literature have investigated the ideal discharge time in very advanced preterm and moderate preterm infants. The aim of this study was to determine the effect of gestational age and accompanying diseases on the ideal discharge time and to ensure that health personnel can provide the most accurate information for parents regarding the ideal discharge time.

## 2. Materials and methods

The study was carried out between January 1, 2016, and May 1, 2020, in Türkiye’s Mersin University Hospital NICU by means of a retrospective file review. The hospital’s NICU has 27 incubators (14 level 4, 13 level 3) and a nurse-patient ratio of 1/3. The study was approved by the institutional clinical research ethics committee (April 28, 2021, 2021/356). Seven hundred nine preterm infants born between gestational weeks 24^0–7^ and 35^0–7^ were evaluated from among 3182 infants born between January 1, 2016 and May 1, 2020. Twenty-eight preterm infants who died were excluded from the study, and the analysis continued with 681 infants. Preterm infants with a gestational age greater than 35^0–7^ weeks and preterm infants with major congenital anomalies were excluded from the study. A flow chart showing patient selection and the methods employed is provided in Figure.

Comorbidities associated with prematurity were defined on the basis of the previous literature. Accordingly, bronchopulmonary dysplasia (BPD) was defined as the persistence of oxygen requirements for at least 28 days in preterm infants with a gestational age <32 weeks and persistence of oxygen requirements at 56 days or at discharge (whichever was earlier) [3]. Gestational age was defined as being between postnatal >28 days and <56 days for preterm infants of >32 weeks, or continued oxygen demand at discharge, whichever is earlier [3]. Patients with suspected necrotizing enterocolitis (NEC) were evaluated based on modified Bell’s staging criteria and were regarded as stage 2 and 3 NEC comorbidity [4]. “Plus” disease at any stage in Zone I, stage 3 retinopathy of prematurity (ROP) in Zone I, stage 2, or stage 3 ROP, and “plus” disease in Zone II, vascular endothelial growth factor (VEGF), and/or laser treatment administration were regarded as comorbidities [5]. The classification developed by Volpe was used for intraventricular hemorrhage (IVH) based on cranial ultrasonography images [6]. Stage 2, 3, and periventricular hemorrhagic infarction were regarded as comorbidities. The presence of two or more of these was regarded as comorbidity.

The primary outcome measure was week of discharge (length of hospital stay + week of birth). The covariables were maternal and infant characteristics likely to affect the duration of hospital stay [7–10]. The ideal discharge time was determined to be 40 weeks according to postmenstrual age (week of birth + length of hospital stay) [11]. The second variable was the effect of the presence or absence of comorbidity affecting the length of hospital stay on discharge at the expected delivery date.

Premature infants are discharged when they meet the criteria. These are that serious medical problems are resolved, apnea and bradycardia are not present, age-appropriate vaccinations have been administered, and infants are able to maintain body temperature in an open bed, and are able to achieve full enteral nutrition. In addition, hearing and heel blood screening tests must be performed, the car seat test must be passed, mother-preterm infant compatibility must be ensured, and the family must be trained to provide the necessary care and treatments.

### 2.1. Statistical analysis

The Shapiro–Wilk test was utilized to check distribution normality for the continuous measurements. The independent samples t test was applied to compare differences between comorbidities and noncomorbidities for continuous measurements. Descriptive statistics were presented as mean ± standard deviation. The Kruskal–Wallis test was applied to determine differences in Apgar scores and mechanical ventilator support parameters depending on gestational age. Minimum and maximum values, median values, and 25%–75% percentages were presented as descriptive statistics. The Mann–Whitney U test was used for binary comparisons. Pearson’s chi-square, Fisher’s exact chi-square, and likelihood ratio chi-square tests were used to analyze differences between categorical variables. The ‘z’ test was used for comparisons between ratios. Corrections were made using the Bonferroni method. p-values <0.05 were regarded as statistically significant.

## 3. Results

Seven hundred nine infants born at 24–35 weeks treated in patient basis in the NICU between January 1, 2016, and May 1, 2020, were identified, with mortality occurring in 28 of these. The 5-year mortality rate was 3.9% in these preterm infants. Exitus infants were removed from the analysis in order to determine the ideal discharge time. The general demographic and clinical characteristics of the 681 infants born between 24 and 35 weeks and classified by gestational age are presented in Table 1. Examination of the demographic data showed that male/female sex distribution at 31^0^–32^0–7^ weeks in both male and female neonates differed compared to the other groups, and that the significant p-value derived from this group (p < 0.005). The most common cause of prematurity at all gestational ages was preterm labor (n: 490, 72%). Other causes included placental anomalies (n:50, 7.4%), preeclampsia/eclampsia (n:43, 6.2%), oligo/polyhydramnios (n:34, 5%), premature rupture of membranes (n:26, 3.8), diabetic mother infant (n:12, 1.8%), and other factors (n:26, 3.8%). The effects of antenatal steroid administration, resuscitation requirement, surfactant administration, sepsis, postnatal dexamethasone therapy, and Apgar scores on the week of discharge (week of birth + hospital stay) were examined. The analysis revealed statistically significant differences (p < 0.001 for all). The use of A T-piece resuscitator (T-PR) in delivery room resuscitation was statistically significant between 24^0–7^ and 25^0–7^–26^0–7^ weeks and 29^0–7^–35^0–7^ weeks (p < 0.05 for all). Requirements for resuscitation with T-PR decreased from gestational weeks 29^0^–30^0–7^ to week 35 (p < 0.05), while an increase was observed at weeks 240, 25^0^–26^0–7^ , and 27^0^–28^0–7^ compared to the other gestational weeks (p < 0.05).

Intubation decreased as the gestational week increased. Differences were observed between 25^0–7^–28^0–7^ weeks and 29^0–7^–35^0–7^ weeks in terms of both intubation and follow-up on a mechanical ventilator: Synchronous intermittent mechanical ventilation + volume guarantee + pressure support ventilation (SIMV-PSV-VG) mode (p < 0.05). Discharge at less than 40 weeks was determined in 87.9% of patients not requiring resuscitation, in 94.7% of those requiring positive pressure ventilation with a T-PR, and in 72.4% of infants requiring intubation (Table 1).

Analysis showed that 74.1% of premature infants who were given exogenous surfactant due to respiratory distress syndrome (RDS) and 81.9% of those who were not given exogenous surfactant were discharged before the expected delivery date. In terms of the relationship between gestational age and the administration of exogenous surfactant, application was more frequent at weeks 24^0^, 25^0^–26^0–7^, 27^0^–28^0–7^, and 29^0^–30^0–7^ compared to the other gestational weeks (p < 0.05 for all). Exogenous surfactant administration increased as gestational age decreased. The differences between gestational age groups and comorbidities (BPD; IVH; ROP and clinical/definite sepsis) are summarized in Table 1. Additionally, 95.1% of preterm infants without BPD were discharged before the expected delivery date (40 weeks). The week of discharge was after the expected delivery week in 64.3% of those infants who received a single course of dexamethasone-furosemide therapy and in 57.1% of those who received two courses of treatment for BPD.

The effect of sepsis on expected delivery week was evaluated. Analysis showed that 77.4% of premature infants with clinical/evidence of sepsis were discharged after the expected week of delivery (PMA 40 weeks), and 97.3% of those without clinical/evidential sepsis were discharged before the expected week of delivery (PMA <40 weeks).

The effect of the presence and absence of comorbidity according to gestational age among the patients in the study group on the ideal discharge time (40 weeks, according to postmenstrual age) was also evaluated (Table 2). Birth weights, discharge weights, hospital stay and discharge times were compared in terms of weeks of gestation (Table 3). Table 3 shows that all infants who were born at gestational week 24^0–7^ were discharged after the expected date of birth. The average LOS of infants born at weeks 25^0–7^–26^0–7^ (99.9 ± 31.2 days in the presence of comorbidity and 68.9 ± 25.7 days in the absence of comorbidity), 27^0–7^–28^0–7^ (LOS 77.8 ± 27.6 days in the presence of comorbidity and 60.2 ± 14.5 days in the absence of comorbidity) and 29^0–7^–30^0–7^ (LOS 53.2 ± 43.7 days in the presence of comorbidity and 33.5 ± 5.9 days in the absence of comorbidity) was significantly shorter than in the absence of comorbidity (p = 0.001, 0.004, and 0.008, respectively; Table 3). Discharge before the expected delivery date was statistically significant if postmenstrual age increased from week 25^0–7^–26^0–7^ and in the absence of comorbidity (p < 0.001). More than half (53.5%) of cases were discharged before the expected date of birth as the gestational week increased, despite the prolonged LOS in the presence of comorbidity (Table 2). In terms of the effect of birth weight on comorbidities, comorbidity was only statistically significant in infants with low birth weight at gestational weeks 25^0–7^–26^0–7^ (p = 0.015). Although infants were discharged at a higher weight in the presence of comorbidity at all weeks, these differences were only statistically significant at weeks 29–30 and 33–34 (p = 0.035 and 0.005, respectively) (Table 3).

## 4. Discussion

The optimal timing of the discharge home of preterm infants is a difficult issue for clinicians. Sepsis, persistent apnea, seizures, and enteral orogastric/nasogastric tube feeding, and prematurity problems such as RDS, BPD, NEC, ROP, and IVH lead to longer hospital stays [12]. Numerous recent innovations have resulted a significant decrease in the frequency of comorbid diseases in premature neonates born at low gestational age, except for BPD. The increased chance of survival among premature neonates born at increasingly lower weeks of gestation has made BPD an inevitable morbidity, due to the pathogenesis of the disease [13]. Stoll et al. [14] classified sepsis and BPD as the two most common causes of morbidity, in line with the increase in survival rates of infants with a gestational age of ≥ 24 weeks. Maier et al. [15] identified BPD, at least one surgical intervention and, to a lesser extent, intracranial hemorrhage/periventricular leukomalacia as the most important risk factors for lengthy hospital stays and not being discharged at the expected date of birth. Similarly to the previous literature, the fact that more than half of the preterm infants treated with dexamethasone for moderate/severe BPD were discharged after the expected delivery date in the present study pinpoints BPD as the most important comorbidity prolonging hospital stay, and thus delaying discharge before the expected delivery date. In addition, 97.3% of preterm infants without sepsis and 77.4% of those with sepsis were discharged before the expected delivery date. This shows that sepsis was another important factor prolonging hospital stay.

Nasal CPAP should be started from birth in premature infants at risk of RDS [16]. The need for both mechanical ventilation and surfactant therapy can thus be reduced through this approach [16]. Cord clamping (30–60 seconds) in our delivery room is performed when spontaneous breathing is present, at low oxygen concentrations, and according to the target oxygen range. Early nasal CPAP is performed with measurable PEEP (5–8 cmH2O). Although antenatal steroid dose was quite low and cesarean section rates were relatively high in this study, it seems clear that these practices contributed to the discharge of 74.1% of premature neonates with RDS before the expected delivery date.

Extremely preterm infants (<28 weeks) are at the highest risk of comorbidity. Seaton et al. [17] found similar lengths of hospital stay and discharge at the expected delivery date among infants born at 24 and 25 weeks of gestation, with hospital stays of approximately 123 and 107 days, respectively, until discharge. However, they also reported that the average LOS of infants born at gestational weeks 30 and 31 was approximately 1 month shorter than the estimated date of birth [17]. Patel et al. [11] calculated the length of hospital stay among 1,337,295 patients born during gestational weeks <24–36, and reported that 74.6% of infants with a gestational age <28 weeks and 98.7% of those over >29 weeks were discharged before the expected date of birth. In the present study, the longest hospital stay was 147 days and the shortest was 119 days among preterm infants surviving from the 24th gestational week to discharge, and BPD and stage 2 IVH were detected in all these. It was, therefore, difficult to conclude that these infants were capable of being discharged on the expected date of birth. At gestational weeks 25^0–7^–26^0–7^, 94% of infants were discharged in the absence of comorbidity, and 53.5% were discharged before the expected week of birth, despite increased hospital stays in the presence of comorbidity. It may therefore be concluded that such preterm infants can be discharged 6 weeks prior to the earliest expected delivery date in the absence of comorbidity, and close to the expected delivery date in the presence of comorbidity. We observed that 96.6% of preterm infants born at 27^0–7^–28^0–7^ weeks of gestation with no comorbidity and 71.1% with comorbidity were discharged before the expected delivery date. These premature infants can be discharged at the 34th week of postmenstrual age at the earliest in the absence of comorbidity, and at the 35th week in the presence of comorbidity.

A study from the United Kingdom examining the cases of 531 infants born at gestational weeks 27^0–7^–33^0–7^ reported that on a ‘Train-to-Home’ package scale 75% were capable of discharge at a postmenstrual age of weeks 36–37 for almost all pregnancies before the expected due date [18]. The data from the present study showed that premature infants born at gestational weeks 29^0–7^–30^0–7^ can be discharged 8 weeks (34.4 ± 1.9) before the earliest expected date of delivery when there are no comorbidities. We concluded that almost all of these (98.5%) were capable of being discharged at 8 weeks at the earliest (34.0 ± 2.1) in the absence of comorbidity at 31^0–7^–32^0–7^ weeks of gestation.

While markers of physiological maturity are an important finding in very extremely preterm and extremely preterm infants during preparation for discharge, their effectiveness decreases in middle and late preterm infants. Although the length of hospital stay of moderate premature infants has been gradually shortened in recent years, parents are still informed that their infants will be discharged home on approximately their estimated date of birth [19,20]. These infants are more likely to develop respiratory distress, heat imbalance, hypoglycemia, kernicterus, apnea, seizures, and nutritional problems compared to infants born on time [21,22]. A community-based study from Sweden evaluated 30–34-week premature infants and reported an average postmenstrual age at discharge of 36.9 ± 1.7 weeks [23]. Authors have reported that high (≥35 years) maternal age, multiple births, being small for gestational age, respiratory distress syndrome, infection, hypoglycemia, and hyperbilirubinemia may be associated with a 13% later discharge, while breastfeeding shortens this period by an average of 2.7 days [23]. In a national, population-based cohort study of late preterm infants, Aly et al. [24] divided 81,913 late preterm infants into two groups 33–34 and 35–36 weeks. Those authors assessed the relationship between demographic and clinical factors in relation to hospital stays, and found that approximately 99% of infants did not remain in hospital for longer than 32 days. Higgins et al. [25] stated that middle and late preterm infants can be discharged at 36 weeks of gestational age. In the present study, the length of the hospital stay decreased due to increased maturity and decreased comorbidity towards moderately preterm (33^0–7^–34^0–7^) and late preterm (35^0–7^) weeks of gestation. The most important reason for hospitalization among the mid-preterm infants (32–35 weeks) in this study was neonatal tachypnea. In addition, preterm infants at 35^0–7^ weeks of gestation had fewer problems requiring hospitalization. The average hospital stay at weeks 33^0–7^– 34^0–7^ was 10.8 ± 8.2 days in the absence of comorbidity and 25.5 ± 16.4 days in the presence of comorbidity, while at weeks 35^0–7^, the average hospital stay was 8.1 ± 6.0 days in the absence of comorbidity. Therefore, since 98% of late preterm infants without comorbidities are discharged before the expected delivery date, health professionals can inform families that these can be discharged at 36 weeks of gestation.

Premature infants are rarely discharged without acquiring the ability to suck and feed [26]. Some studies have suggested that early discharge can be safe if physiological criteria (growth, temperature protection, and control of breathing) are met instead of discharge weight or expected date of birth in discharge before the expected date of birth [27]. A study from India assessed the early discharge of premature (26–34 weeks) infants due to limited bed capacity in terms of patient requirements in the NICU. The authors concluded that infants born earlier than 34 weeks can be safely discharged when they meet the physiological development criteria [28]. Merritt et al. [29] reviewed professional guidelines and standards of care and noted that early discharge is possible despite differences in neonatal care practices and LOS. The findings of the present study showed that comorbidity is the most important criterion in the discharge decision.

The principal limitations of this study are that it was based on a single center, and the data were obtained from file data defined with retrospective ICD codes. Another limitation involved the innovations in our treatment and care practices that have taken place over the years (such as minimally invasive surfactant application as the first-choice option in the last three years, and cord clamping time [30–60 s] planning). We believe that these changes over the years may have affected the study results. The distinct strength of this study is that our single-center results represent an important step towards understanding when preterm infants at 24–35 weeks can be discharged in the presence and absence of comorbidity.

## 5. Conclusions

Healthcare professionals wish to know the most reliable discharge timing estimate for preterm infants to be able to inform families accordingly. Professionals can inform families that, in the absence of comorbidity, discharge is possible at an average of 36 weeks for 25–28-week gestational ages, and at an average of 34 weeks for 29–32-week gestational ages. The data presented here will represent a useful guide for healthcare professionals in terms of the ideal discharge time for preterm infant born at or under 35 weeks. Standardized guides can be created for the ideal discharge time through future studies on this subject.

## Figures and Tables

**Figure:**
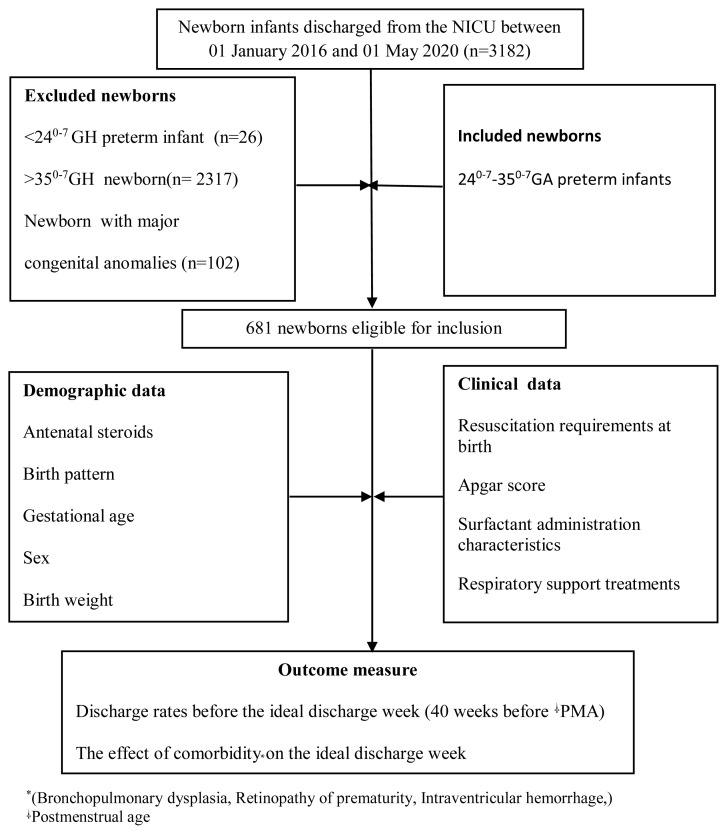
Flow chart of patient selection and methods employed

**Table 1:** Demographic data of the patients in the study.

n		24^0^ week		25^0^–26^0–7^ weeks		27^0^–28^0–7^weeks		29^0^–30^0–7^ weeks		31^0^–32^0–7^ weeks		33^0^–34^0–7^ weeks		35^0–7^ week		p
		%	n	%	n	%	n	%	n	%	n	%	n	%		
Antenatal steroid administration	None/Unknown (n=207)	0	0.0	12	20.0	11	16.4	38	31.7	44	27.8	78	37.1	24	38.1	**0.005**
	One dose (n=170)	1	33.3	22	36.7	13	19.4	23	19.2	41	25.9	53^c^	25.2	17^c^	27.0	
	Two doses (n=304)	2	66.7	26	43.3	43	64.2	59	49.2	73	46.2	79^c^	37.6	22^c^	34.9	
Mode of delivery	Vaginal Delivery (n=35)	0	0.0	3	5.0	1	1.5	5	4.2	13	8.2	13	6.2	0^e^,^f^	0.0	**0.046**
	Cesarean (n=646)	3	100.0	57	95.0	66	98.5	115	95.8	145	91.8	197	93.8	63^e^,^f^	100.0	
Resuscitation requirement	None (n=58)	0	0.0	2	3.3	0	0.0	8	6.7	13	8.2	25	11.9	10^c^	15.9	**<0.001**
	‘’PBV-t piece (n=525)	0	0.0	31	51.7	40	59.7	94^a^,^b^	78.3	135 a,b	85.4	175 a,b	83.3	50 a,b	79.4	
	Intubation (n=98)	3	100.0	27	45.0	27	40.3	18 a,b,c	15.0	10 a,b,c	6.3	10 a,b,c,d	4.8	3 a,b,c	4.8	
Sex	Female (n=58)	2	66.7	34	56.7	28	41.8	69	57.5	58^d^	36.7	98	46.7	32	50.8	**0.015**
	Male (n=360)	1	33.3	26	43.3	39	58.2	51	42.5	100 d	63.3	112	53.3	31	49.2	
SIMV-PS-VG*	Available (n=152)	3	100.0	38	63.3	42	62.7	29^b^,^c^	24.2	19 b,c	12.0	16 b,c,d	7.6	5 b,c	7.9	**<0.001**
	None (n=529)	0	0.0	22	36.7	25	37.3	91 b,c	75.8	139 b,c	88.0	194 b,c,d	92.4	58 b,c	92.1	
HFO Ɨ	Available (n=62)	2	66.7	20	33.3	13	19.4	14^b^	11.7	4^a^,^b^,^c^,^d^	2.5	7 a,b,c	3.3	2 a,b	3.2	**<0.001**
	None (n=619)	1	33.3	40	66.7	54	80.6	106^b^	88.3	154 a,b,c,d	97.5	203 a,b,c	96.7	61 a,b	96.8	
Surfactant administration	Available (n=151)	3	100.0	38	63.3	42	62.7	36^b^,^c^	30.0	13 b,c,d	8.2	15 b,c,d	7.1	4 b,c,d	6.3	**<0.001**
	None (n=530)	0	0.0	22	36.7	25	37.3	84 b,c	70.0	145 b,c,d	91.8	195 b,c,d	92.9	59^b^,^c^,^d^	93.7	
Surfactant administration dose	One dose (n=87)	2	66.7	17	44.7	22	52.4	23	63.9	9	69.2	10	66.7	4	100.0	0.246
	Two doses (n=64)	1	33.3	21	55.3	20	47.6	13	36.1	4	30.8	5	33.3	0	0.0	
Necrotizing enterocolitis	None (n=670)	3	100.0	56	93.3	66	98.5	117	97.5	157	99.4	208	99.0	63	100.0	0.124
	Stage 2 and Stage 3 NEC (n=11)	0	0.0	4	6.7	1	1.5	3	2.5	1	0.6	2	1.0	0	0.0	
Bronchopulmonary Dysplasia(BPD)	Available (n=60)	3	100.0	31	51.7	17^b^	25.4	5^a^,^b^,^d^	4.2	4 a,b,d	2.5	0 a,b,d	0.0	0 a,b,d	0.0	**<0.001**
	None (n=621)	0	0.0	29	48.3	50^b^	74.6	115 a,b,d	95.8	154 a,b,d	97.5	210 a,b,d	100.0	63 a,b,d	100.0	
Number of dexamethasone-furosemide treatment/cures for BPD	No cure (n=632)	0	0.0	33a	55.0	54^a^,^b^	80.6	117^a^,^b^, ^c^	97.5	156^a^,^b^,^c^	98.7	209^a^,^b^, ^c^	99.5	63^a^,^b^,^c^	100.0	**<0.001**
	Single cure(n=42)	2	66.7	24	40.0	11	16.4	3 a,b,c	2.5	1 a,b,c	0.6	1 a,b,c	0.5	0 a,b,c	0.0	
	Two cure (n=7)	1	33.3	3	5.0	2	3.0	0^a^,^b^	0.0	1^a^,^b^	0.6	0^a^,^b^	0.0	0^a^,^b^	0.0	
Retinopathy of Prematurity	None (n=666)	2	66.7	50	83.3	65	97.0	118^b^,^c^	98.3	158 b,c	100.0	210 b,c	100.0	63 b,c	100.0	**<0.001**
	¥VEGF (n=8)	0	0.0	4	6.7	2	3.0	2	1.7	0^b^	0.0	0^b^	0.0	0^b^	0.0	
	Laser (n=7)	1	33.3	6	10.0	0^a^,^b^	0.0	0 a,b	0.0	0 a,b	0.0	0 a,b	0.0	0 a,b	0.0	
Intracranial hemorrhage	None (n=661)	0	0.0	55^a^	91.7	61 a,b	91.0	116 a,b	96.7	157 a,b	99.4	209 a,b	99.5	63 a,b	100.0	**<0.001**
	Stage 2 and/or 3 hemorrhage (n=20)	3	100.0	5^a^	8.3	6 a,b	9.0	4 a,b	3.3	1 a,b	0.6	1 a,b	0.5	0 a,b	0.0	
clinical/proven sepsis	None (n=513)	0	0.0	22	36.7	37	55.2	78^a^,^b^	65.0	139^a^,^b^,^c^	88.0	183^a^,^b^,^c^	87.1	54^a^,^b^		<0.001
	clinical/proven sepsis (n=168)	3	100.0	38	63.3	30	44.8	42^a^,^b^	35.0	19^a^,^b^,^c^	12.0	27^a^,^b^,^c^	12.9	9^a^,^b^		

“PBV: Positive pressure ventilation,

* SIMV-VG-PSV; Synchronous intermittent mechanical ventilation + volume guarantee + pressure support ventilation,

Ɨ: HFO: High frequency oscillation,

¥ VEGF; vascular endothelial growth factor; It shows the differences with

a 24,

b 25–26,

c 27–28,

d 29–30,

e 31–32 and

f 33–34 weeks.

**Table 2:** Expected discharge rates at time of birth depending on comorbidity.

Gestational week	Discharge time	Comorbidity present		No comorbidity		p
		Number	Percentage	Number	Percentage	
24^0–7^ week	Less than 40 weeks (n=0)	0	0.0	0	0.0	-
	40 weeks and above (n=3)	3	100.0	0	0.0	
25^0–^-26^0-7^ weeks	Less than 40 weeks (n=39)	23	53.5	16	94.1	**0.003**
	40 weeks and above(n=21)	20	46.5	1	5.9	
27^0^–28^0-7^ weeks	Less than 40 weeks (n=55)	27	71.1	28	96.6	**0.009**
	40 weeks and above(n=12)	11	28.9	1	3.4	
29^0^–30^0-7^ weeks	Less than 40 weeks(n=110)	33	80.5	77	97.5	**0.003**
	40 weeks and above (n=10)	8	19.5	2	2.5	
31^0^–32^0-7^ weeks	Less than 40 weeks (n=154)	22	91.7	132	98.5	0.110
	40 weeks and above (n=4)	2	8.3	2	1.5	
33^0^–34^0-7^ weeks	Less than 40 weeks (n=201)	24	80.0	177	98.3	**<0.001**
	40 weeks and above (n=9)	6	20.0	3	1.7	
35^0-7^ week	Less than 40 weeks (n=60)	10	83.3	50	98.0	0.090
	40 weeks and above (n=3)	2	16.7	1	2.0	

**Table 3:** Comparison of comorbidities according to gestational age.

		24^0–7^ week (n=3)	25^0–7^–26^0–7^ weeks (n=60)	27^0–7^–28^0–7^ weeks (n=67)	29^0–7^–30^0–7^ weeks (n=120)	31^0–7^–32^0–7^ weeks (n=158)	33^0–7^–34^0–7^ weeks (n=210)	35^0–7^ weeks (n=63)
Birth weight (g)	¥CP	635.0 ± 65.0	831.6 ± 140.3	1019.3 ± 244.6	1374.9 ± 266.3	1624.5 ± 422.6	1954.0 ± 456.9	2154.6 ± 645.3
	#NC	-	939.1 ± 173.5	1094.2 ± 248.5	1397.8 ± 293.0	1733.7 ± 327.0	2014.7 ± 410.2	2247.1 ± 427.1
	P	-	**0.015**	0.222	0.677	0.152	0.461	0.644
Discharge weight (g)	CP	3176.7 ± 241.7	2578.6 ± 800.1	2309.7 ± 559.2	2418.9 ± 1302.2	2077.9 ± 788.5	2263.8 ± 347.4	2290.0 ± 577.7
	NC	-	2261.2 ± 535.3	2180.0 ± 359.9	1967.6 ± 382.7	1877.9 ± 266.9	2077.9 ± 328.9	2233.2 ± 367.2
	P	-	0.138	0.281	**0.035**	0.231	**0.005**	0.750
*DHS	CP	133.7 ± 16.2	99.9 ± 31.2	77.8 ± 27.6	53.2 ± 43.7	32.1 ± 35.4	25.5 ± 16.4	22.0 ± 16.8
	NC	-	68.9 ± 25.7	60.2 ± 14.5	33.5 ± 5.9	16.7 ± 12.9	10.8 ± 8.2	8.1 ± 6.0
	P	-	**0.001**	**0.001**	**0.008**	**0.046**	**<0.001**	**0.016**
Discharge	CP	43.7 ± 2.3	39.3 ± 4.6	38.2 ± 3.5	37.2 ± 6.2	36.0 ± 5.0	35.4 ± 6.7	37.0 ± 2.1
	NC	-	36.0 ± 2.7	36.2 ± 2.0	34.4 ± 1.9	34.0 ± 2.1	34.9 ± 1.3	36.0 ± 0.9
	P	-	**0.001**	**0.004**	**0.008**	0.062	0.685	0.126

* Day of Hospital stay,

¥ Comorbidity present,

# No comorbidity

## Data Availability

The data that support the findings of this study are available from the corresponding author upon reasonable request.
